# Genetic manipulation resulting in decreased donor chondroitin sulfate synthesis mitigates hepatic GVHD via suppression of T cell activity

**DOI:** 10.1038/s41598-023-40367-3

**Published:** 2023-08-11

**Authors:** Suguru Tamura, Hajime Ishiguro, Tatsuya Suwabe, Takayuki Katagiri, Kaori Cho, Kyoko Fuse, Yasuhiko Shibasaki, Tadahisa Mikami, Takero Shindo, Hiroshi Kitagawa, Michihiro Igarashi, Hirohito Sone, Masayoshi Masuko, Takashi Ushiki

**Affiliations:** 1 Department of Hematology, Niigata University Faculty of Medicine, 1-757 Asahimachi-dori, Chuo-ku, Niigata City, Niigata 951-8510 Japan; 2 Laboratory of Biochemistry, Kobe Pharmaceutical University, Kobe, Japan; 3 Department of Hematology/Oncology, Kyoto University Graduate School of Medicine, Kyoto, Japan; 4 Department of Neurochemistry and Molecular Cell Biology, Niigata University Graduate School of Medical and Dental Sciences, Niigata, Japan; 5 Division of Hematology and Oncology, Graduate School of Health Sciences, Niigata University, Niigata, Japan

**Keywords:** Biomarkers, Medical research

## Abstract

Donor T cell activation, proliferation, differentiation, and migration are the major steps involved in graft-versus-host disease (GVHD) development following bone marrow transplantation. Chondroitin sulfate (CS) proteoglycan is a major component of the extracellular matrix and causes immune modulation by interacting with cell growth factors and inducing cell adhesion. However, its precise effects on immune function are unclear than those of other proteoglycan families. Thus, we investigated the significance of CS within donor cells in acute GVHD development utilizing CSGalNAc T1-knockout (T1KO) mice. To determine the effects of T1KO, the mice underwent allogenic bone marrow transplantation from major histocompatibility complex-mismatched donors. While transplantation resulted in hepatic GVHD with inflammatory cell infiltration of both CD4^+^ and CD8^+^ effector memory T cells, transplantation in T1KO-donors showed milder cell infiltration and improved survival with fewer splenic effector T cells. In vitro T-cell analyses showed that the ratio of effector memory T cells was significantly lower via phorbol myristate acetate/ionomycin stimulation. Moreover, quantitative PCR analyses showed significantly less production of inflammatory cytokines, such as IFN-γ and CCL-2, in splenocytes of T1KO mice. These results suggest that reduction of CS in donor blood cells may suppress the severity of acute GVHD after hematopoietic stem cell transplantation.

## Introduction

Severe graft-versus-host disease (GVHD) is a major complication that develops after allogeneic hematopoietic stem cell transplantation. The etiology of acute and progressive GVHD is primarily the response of donor T cells to allo-antigens of the recipient^1^. On the basis of experimental models, the development of acute GVHD can be conceptualized in three sequential steps or phases: (1) activation of the antigen presenting cells (APCs) by the damaged host tissues where the levels of proinflammatory cytokines, chemokines, and adhesion molecules increase; (2) donor T cell activation, proliferation, differentiation, and migration; and (3) target tissue destruction^2^.

**Figure 1:**
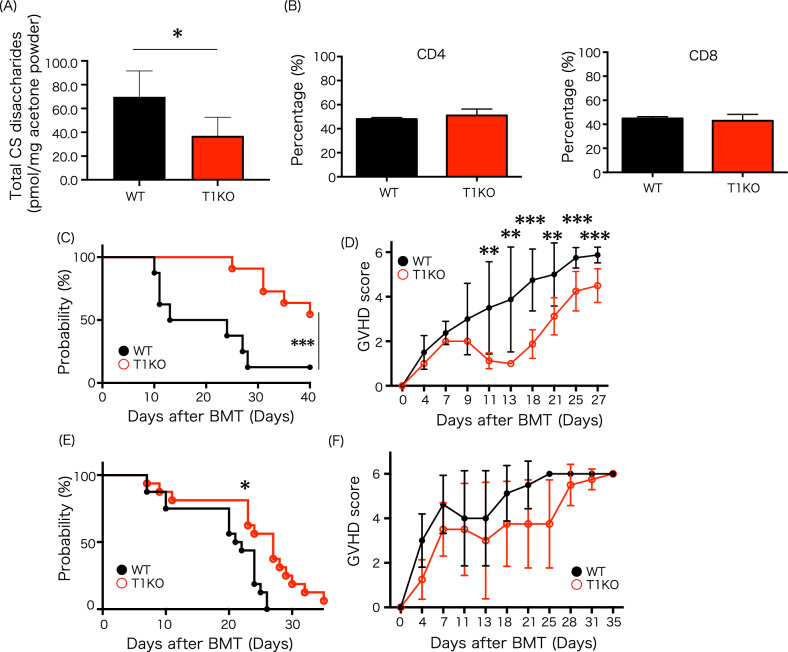
Decreased donor CS mitigates GVHD. (**A**) The total splenic CS disaccharides in 8- to 12-week-old WT mice and T1 KO mice analyzed by HPLC-based quantification assay (WT, n = 6; T1KO, n = 6). Samples were collected five times and measurements were made once. (**B**) T cell subset of transplanted splenocytes, n = 4–5 mice per group. The experiment was performed five times. (**C**,**D**) Overall survival and GVHD scores after BMT in mice with lacking T1 enzyme and WT. (**C**) overall survival (WT, n = 8; T1KO, n = 11) and (**D**) GVHD scores (WT, n = 8; T1KO, n = 8) after 5 × 10^6^ BM-MNC with whole splenocytes (including 1 × 10^6^) transplantation. Transplantation was performed five times. (**E**) overall survival (WT, n = 12; T1KO, n = 16) and (**F**) GVHD scores (WT, n = 8; T1KO, n = 8) after 5 × 10^6^ BM-MNC with 3 × 10^6^ CD90.2 + cell transplantation. Transplantation was performed five times. *p < 0.05, **p < 0.01, and ***p < 0.001 for pairwise comparison of survival of T1KO and *WT* Mantel-Cox Log-rank test.

The extracellular matrix (ECM) not only provides tissue support, but its functional macromolecules also regulate cell properties and function via interactions between ECM components and cell-surface receptors, growth factors, and cytokines^3^. Proteoglycans (PGs) are present in almost all tissues at the extracellular and cellular (cell membrane and intracellular) levels^3^. PGs consist of core protein and glycosaminoglycan (GAG)^4^ (Supple Fig. 1). GAG chains are linear polysaccharides with repeating disaccharide sequences and are synthesized by more than 10 glycosyltransferases and multiple sulfotransferases^5^. Additionally, GAGs are mainly classified into chondroitin sulfate (CS), hyaluronic acid (HA), dermatan sulfate, heparan sulfate (HS), dermatan sulfate, heparin, and keratan sulfate according to the type of repeating disaccharide unit.

GAGs are expressed in the form of PGs on the surface of T cells^6^ or other immune cells, and regulate T cell function. In fact, extracellular HS can activate Toll-like receptor 4 (TLR4) on dendritic cells. Furthermore, it has been reported that serum levels of HS, a TLR4 agonist, are highly elevated during the onset of acute GVHD after allo-HSCT^7^. Meanwhile, CS is known as a critical mediator of the failure of inflammation resolution after spinal cord injury. Analysis of phenotypically distinct immune cell clusters revealed CS-mediated modulation of macrophage and microglial subtypes, which together with T lymphocyte infiltration and composition changes, suggests a role in modulating both innate and adaptive immune responses after a spinal cord injury^8^. Hence, CS, which is a major subtype of sulfated GAGs, plays an important role in modulating immunity. In addition, CS competitively inhibits the binding of HA to CD44, an HA receptor expressed on the surface of lymphocytes^9^. Binding of CD44 to HA increased in proportion to T cell activation^10^, while reduced connectivity reduces the severity of skin GVHD^11^.

Taken together, PGs may be associated with mechanisms of GVHD via direct or indirect T cell immune modulation; however, to date, the correlation between CS and GVHD has not been fully elucidated. In this study, we used knockout (KO) mice of CSGalNAcT1 (T1), an enzyme required at the first unique step of CS synthesis (Supple Fig. 1). It has been shown that CS synthesis is reduced in cartilage^12^, nerve cells^13–15^, and bone marrow cell^16^ by 30 − 50% in T1KO mice compared to that in wild type (WT) mice. In hematological analyses, the CS disaccharides of bone marrow cells were decreased^16^. However, the role of CS in vivo is unknown, except in the steady state, and its effects on immunity are also unclear.

Herein, we investigated the effects of reduced CS on GVHD using T1KO mice, and showed that low CS in donor cells alleviated GVHD after allogeneic hematopoietic stem cell transplantation via suppression of T cell activity.

## Results

### Decreased donor CS mitigates GVHD

We investigated the effect of CS depletion in donor cells in a mouse model of GVHD. We previously reported that the BM levels of CS disaccharides in T1KO mice were approximately two-thirds of those in WT mice^16^. Since splenic transplantation was also performed this time, CS quantification of splenocytes was also analyzed. As a result, splenocyte CS decreased to 50% of WT in T1KO (Fig. 1A). In addition, there was no significant difference in the CD4/CD8 cell ratio in the spleens of donor T1KO and WT mice (Fig. 1B). First, GVHD was induced by whole spleen mononuclear cells (SpMNCs) transplantation. The median survival time was clearly prolonged in the group transplanted with the T1KO donor T cells compared to the WT donor T cells (Fig. 1C). Moreover, the GVHD score from day 13 to 27 was significantly reduced in T1KO group (Fig. 1D). Thereafter, to determine whether the number of CD90.2^+^ T cells transplanted affected the reduction of GVHD, GVHD was induced by CD90.2^+^ T cells, our results demonstrated that T1KO donor T cells significantly improved overall survival (OS) compared to that by WT donor T cells (21.50 days versus 27.00 days, *p* < 0.01) (Fig. 1E). GVHD scores decreased, but no significant difference was noted (Fig. 1F). Taken together, reduction of CS in donor cells attenuated GVHD and prolonged OS. Furthermore, we revealed that the amount of transplanted donor T cells induced the relief of GVHD.

### The mechanism of hepatic GVHD mitigation

**Figure 2:**
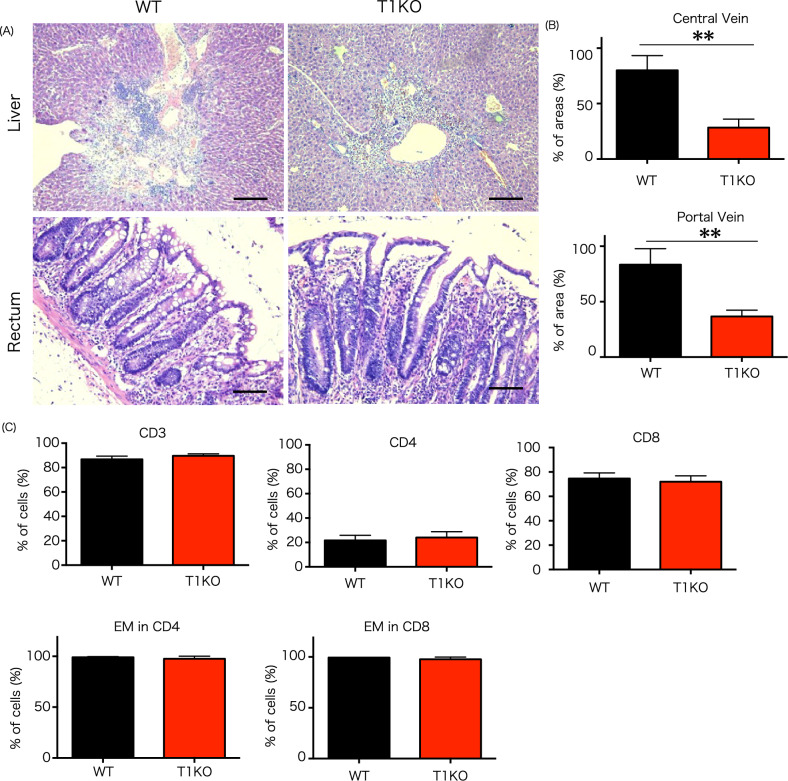
Features of pathology and infiltration markers of donor cells in the liver. (**A**) Photomicrograph (hematoxylin and eosin stain) showing inflammation and mixed hematopoietic infiltration of the liver and rectum in T1KO mice at day 28 following BMT, n = 3 in each group. Scale bar = 100 μm. Data were taken from five independent experiments. (**B**) Counting hepatic infiltration at day 28 following BMT. Twenty independent areas were counted around the central vein and portal area per mouse. n = 3 in each group. The experiment was performed five times. (**C**) Representative flow cytometry profiles of CD4 and CD8 effector memory T cells in the liver at day 28 following BMT. n = 3 in each group. The experiment was performed four times. Mean ± SD is shown with **p < 0.01 for comparison, unpaired Student *t* tests. *EM* effector memory T cell.

Euthanization was performed on day 28 after allogeneic hematopoietic stem cell transplantation in the intermediate group, and histological evaluation was performed on the liver, skin, and intestinal tract (rectum) (Fig. 2A). Liver pathology revealed that hepatic GVHD was observed in both the portal and central vein regions in the WT group, and showed that cell infiltration was reduced in T1KO donor T cell group. In addition, the infiltration area decreased in T1KO donor T cell group compared to that in WT donor T cell group (Fig. 2B). Furthermore, on day 28 post-transplantation, infiltrating cells in the liver were examined via flow cytometry (Fig. 2C), and displayed no difference in the CD4/CD8 ratio between the two groups, whereby both CD4 and CD8 are composed of CD44^+^CD62L^-^ effector memory T cells.

### T lymphocyte proliferative capacity in T1KO

**Figure 3:**
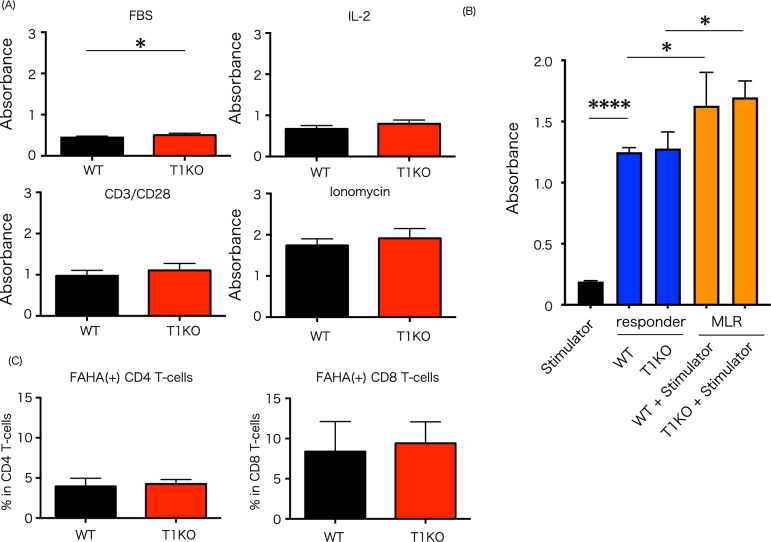
Mixed lymphoid reaction and HA binding assay in T1KO mice. (**A**) WST-8 assay, n = 6 in each group. The experiment was performed twice. (**B**) Mixed lymphoid reaction. Stimulator and responder were cultured for 7 days. Stimulator: Balb/c SpMNCs; Responder: T1KO CD90.2^+^ T cells. n = 3 in each group. The experiment was performed thrice. (**C**) HA binding assay, n = 3 in each group. The experiment was performed twice. Mean ± SD is shown with *p < 0.05 and ****p < 0.0001 for comparison, unpaired Student *t* tests (**A**,**C**) and One-way ANOVA with Tukey’s multiple comparisons test (**B**).

SpMNCs were cultured with IL-2 for 48 h to examine the proliferation of T cells. IL-2 stimulation significantly increased the proliferation of T1KO donor T cells compared to that of WT donor T cells. However, stimulation with CD3 or phorbol myristate acetate (PMA)/ionomycin did not change proliferation between T1KO and WT donor T cells (Fig. 3A). No difference in cell proliferation was observed between WT and T1KO donor splenocytes in the mixed (Supple. Fig. 2). More cautiously, MLR was performed using T1KO CD90.2 cells as responder, but there was no difference in the results from WT (Fig. 3B).

### Binding capacity between T1KO lymphocytes and HA

CS inhibits the binding of CD44 donor cells to HA. In addition, since the liver contains a large amount of hyaluronan in the extracellular matrix, which is associated in the mechanism that exacerbates hepatic GVHD, we evaluated the ability of T1KO splenocytes to bind to hyaluronan. After stimulating SpMNCs with PMA/ionomycin for 48 h, we compared the binding rates of fluorescence-labeled HA (FAHA) and CD44 (Fig. 3C); our results displayed no significant difference in either the CD4 or CD8 groups.

### Apoptosis among T lymphocytes in T1KO group

**Figure 4:**
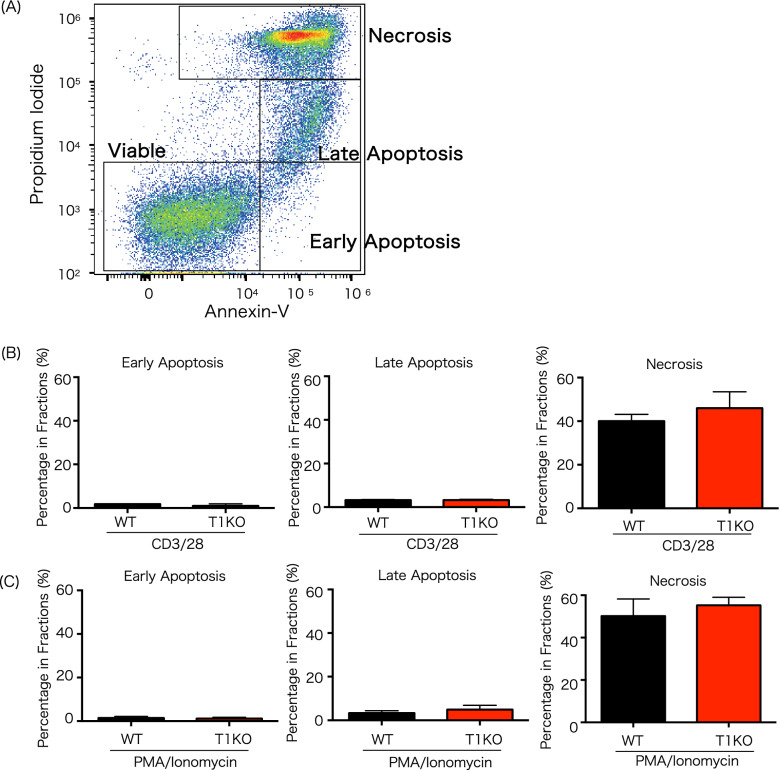
Apoptosis assay on T1KO mice in vitro. (**A**) Apoptosis/necrosis was classified indicated gating. The percentage of early apoptotic (Annexin V^+^ Propidium Iodide^−^) and late apoptotic cells (Annexin V^+^ Propidium Iodide^+^) is shown. Percentage of early/late apoptotic cell and necrosis ratio 48 h after stimulation with (**B**) anti-CD3/28 or (**C**) PMA/ionomycin, n = 7 in each group. The experiment was performed four times. Mean ± SD is shown, unpaired Student *t* tests.

FACS analysis using Annexin-V and Propidium Iodide compared apoptosis after T cell stimulation with CD3/CD28 beads or PMA/ionomycin (Fig. 4). No significant difference was found in the proportion of apoptotic cells under any condition. Moreover, it was classified into early apoptosis and late apoptosis according to the expression intensity of Propidium Iodide. No significant difference was found in either early or late apoptosis.

### Impaired activation of CD4 and CD8 lymphocytes of T1KO mice

**Figure 5:**
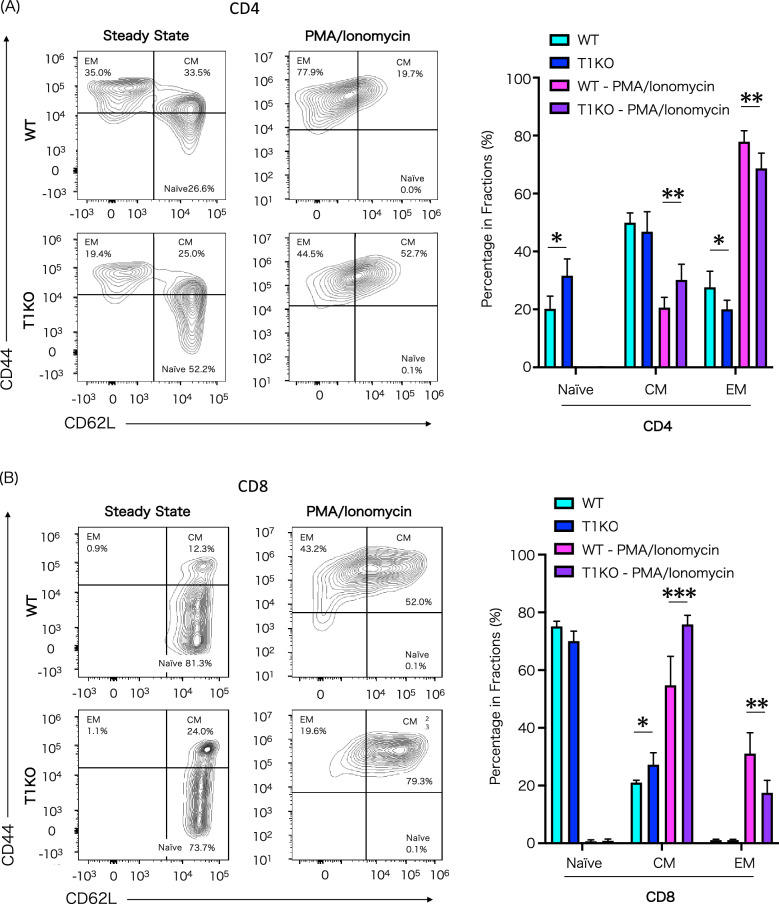
T cell subset in T1KO mice after stimulation in vitro. Proportions of naïve (CD44^-^CD62L^+^), central memory (CD44^+^CD62L^+^), and effector memory (CD44^+^CD62L^−^) T cells in SpMNCs at 48 h after stimulation with PMA/ionomycin. (**A**) Proportions of CD4 T-cell subset before and after culture is shown. (**B**) Proportions of CD8 T-cell subset before and after culture is shown. n = 4 − 6 in each group. The experiment was performed five times. Mean ± SD is shown with *p < 0.05, **p < 0.01, and ***p < 0.001 for comparison, One-way ANOVA with Tukey’s multiple comparisons test. *CM* central memory T cell, *EM* effector memory T cell.

Splenic T cells were analyzed for T cell subsets (Fig. 5). In the steady state before stimulation, the ratio of naïve cells was significantly higher in T1KO mice than in WT mice, and the ratio of effector memory cells was significantly lower in T1KO mice, indicating impaired functional differentiation of CD4 T cells. Simultaneously, almost no effector memory cells were observed in the groups. Moreover, CD90.2^+^ T cells were stimulated with PMA/ionomycin for 48 h. No differences were found in the ratios of CD4 and CD8 cells. Additionally, functional differentiation into CD4 and CD8 effector memory T cells with stimulation was markedly impaired in T1KO mice (Fig. 5A,B). Furthermore, lymphocyte co-stimulatory factors, analyzed by T cell stimulation with PMA/ionomycin and PD-1, which is a T cell activation marker, were expressed at a higher level in WT mice than in T1KO mice (Supple. Fig. 3). Other co-stimulatory factor expressions, such as CD49d, KLRG-1, LFA-1, and LPAM-1, showed no clear modulation.

### Splenocytes from T1KO mice show reduced production of inflammatory cytokines and chemokines

**Figure 6:**
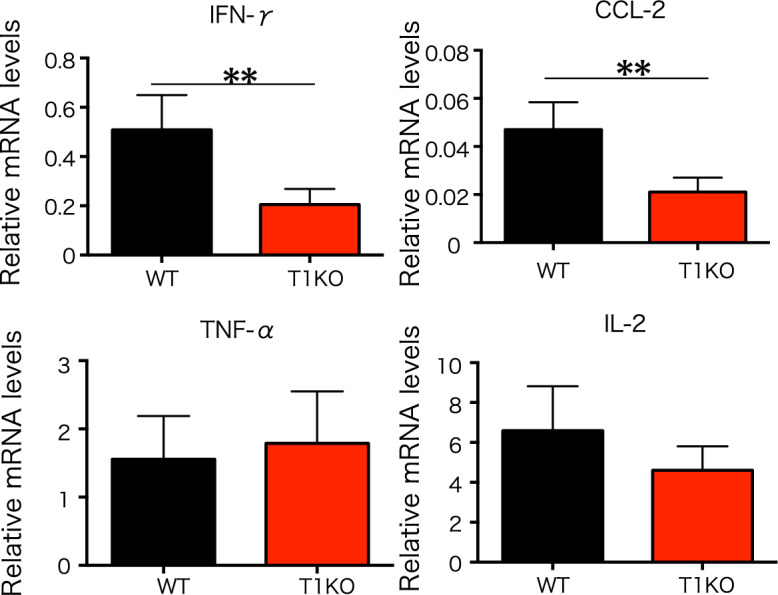
Features of inflammation and infiltration markers of T cells in vitro. qPCR expressions on SpMNCs at 48 h after stimulation with PMA/ionomycin. Inflammation marker: IFN-γ, TNF-α, and IL-2 mRNA levels; infiltration marker: CCL-2 mRNA levels. n = 3 − 4 in each group. The experiment was performed three times. Mean ± SD is shown with **p < 0.01 for comparison, unpaired Student *t* tests.

SpMNCs were stimulated with PMA/ionomycin for 48 h. RT-PCR of SpMNCs was performed. Expressions of inflammatory cytokines, TNFα, and IL-2 did not differ between WT and T1KO groups. However, the expressions of inflammatory cytokine IFN-γ and the chemokine CCL-2 were significantly reduced in T1KO group (Fig. 6).

### Effector memory T cells decrease in T1KO mice after BMT

**Figure 7:**
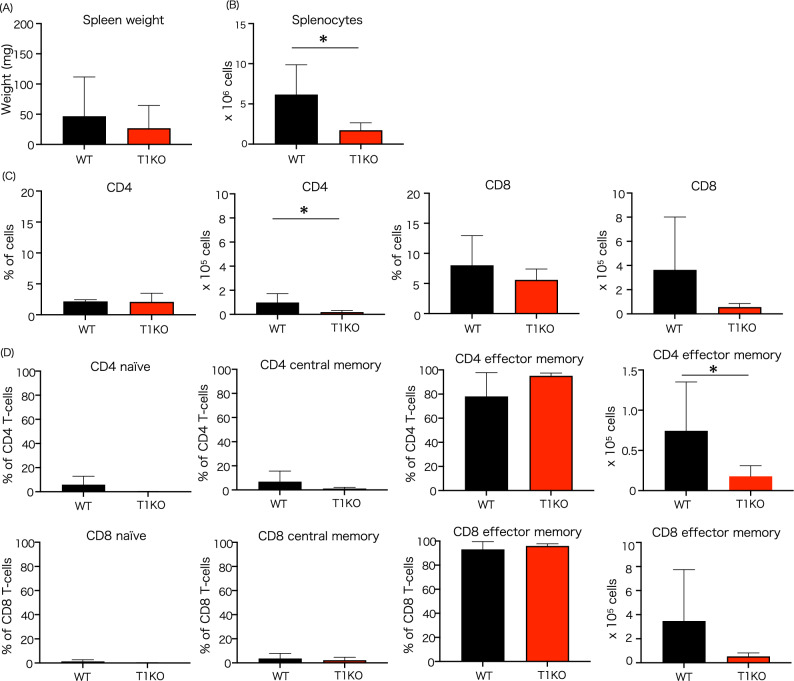
T cell subset in T1KO mice after BMT. Proportions of naïve, central memory, and effector memory T cells in the spleen at day 28 after allogenic BMT. (**A**) Splenic weight and (**B**) cell count at day 28 after BMT. n = 4 − 5 in each group. The experiment was performed twice. (**C**) Proportions of CD4 T-cell subset and (**D**) Proportions of CD8 T-cell subset at day 28 after BMT is shown. n = 5 − 6 in each group. The experiment was performed four times. Mean ± SD is shown with *p < 0.05 for comparison, unpaired Student *t* tests.

T cell subsets in the spleen were observed 28 days after allogenic BMT (Fig. 7). Although there was no significant difference in spleen weight, splenocyte numbers were significantly reduced in T1KO mice (Fig. 7A,B). Therefore, the number of CD4 T lymphocytes in the spleen was significantly decreased, and that of CD8 T cells also tended to decrease (Fig. 7C). Regarding the activation of T lymphocytes, effector memory T cells were the majority. In T1KO, the number of CD4 effector memory T cells decreased significantly, and that of CD8 memory T cells tended to decrease (Fig. 7D).

### Immunomodulating cells and apoptosis in the spleen in T1KO mice after BMT

**Figure 8:**
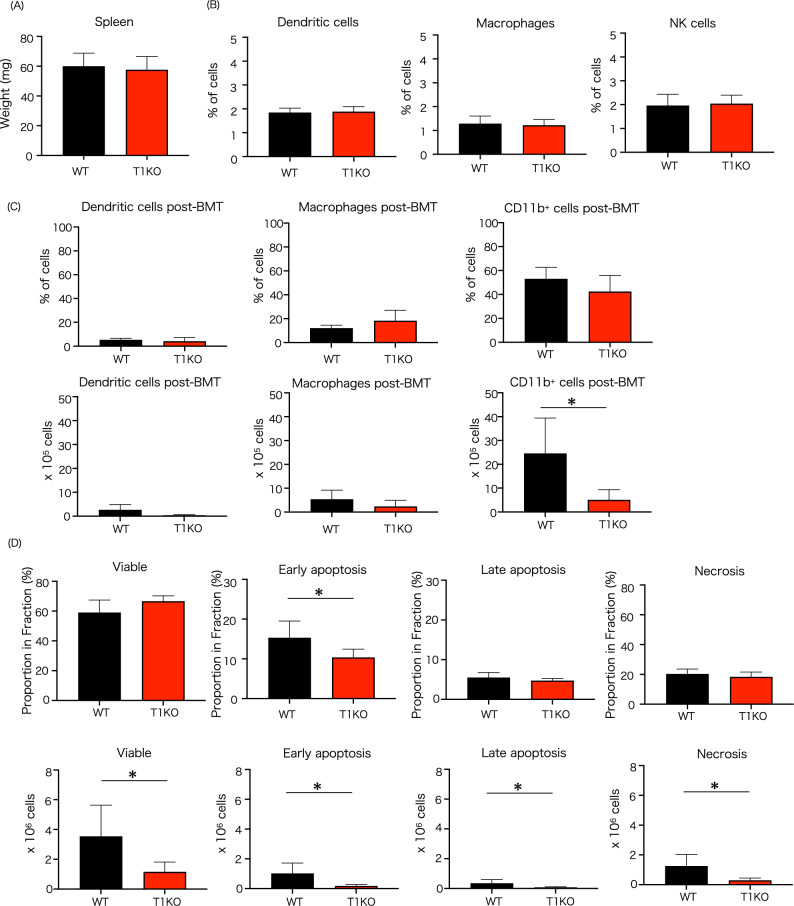
Immune cells excluding T cells and apoptotic state of splenocytes in vivo. Dendritic cells (CD11c^+^), macrophages (CD11b^+^F4/80^+^CD11c^−^), CD11b^+^ cells (CD11b^+^CD11c^−^) and NK cells (CD3^−^NK1.1^+^ CD49b^+)^ subset before and after BMT are shown. (**A**) spleen weight before BMT. (**B**) cell-subset before BMT and (**C**) at day 28 after BMT. (**D**) Apoptotic state at day 28 after BMT. n = 5 − 7 in each group. The experiment was performed four times. Mean ± SD is shown with *p < 0.05 for comparison, unpaired Student *t* tests.

Spleen weight at steady state is unchanged in T1KO mice (Fig. 8A). Further, the ratio of Dendritic cells (CD11c^+^), macrophages (CD11b^+^F4/80^+^CD11c^–^), CD11b^+^ cells (CD11b^+^CD11c^–^) and NK cells (CD3^-^NK1.1^+^ CD49b^+)^ subset were not changed between WT and T1KO before BMT (Fig. 8B). On the 28th day after transplantation, CD11b + myeloid cells in the spleen were the main components. They were significantly decreased in T1KO mice. However, no significant difference was observed for macrophages. There was no change in the number of dendritic cells. Also, the ratio of NK cells was reduced after BMT to almost undetectable levels (Fig. 8C). The percentage of early apoptosis decreased in T1KO, and that of viable cells tended to increase. Further, the number of apoptotic cells was decreased in T1KO mice, reflecting the number of splenocytes (Fig. 8D).

## Discussion

Severity of hepatic GVHD after allogenic BMT was attenuated and OS was prolonged in T1KO mice. To assess the effects of T-cells in these phenomena, we performed GVHD induction by CD90.2 + T cells and confirmed GVHD reduction in T1KO mice. Our results suggest that CS depletion in donor T cells plays an important role in alleviating the severity of GVHD. In the current study, T cell-transplantation was used for GVHD development, the number of effector memory cells decreased and naïve T cells increased in CD4 cells at steady state in the transplanted splenocytes. In addition, central memory T cells were increased in T1KO mice, however, the ratio of effector memory cells was unchanged compared to that in CD8 cells of WT mice at steady state. Hence, we deduced that T cells from T1KO mice are less able in inducing GVHD than those of WT mice. Furthermore, activation and differentiation defects were also observed in T cells from T1KO mice, whereby effector memory cell activation was significantly lower in T1KO mice after PMA/ionomycin stimulation. CS is necessary to maintain the diversity and differentiation potential of embryonic stem cells, and without CS, only self-renewal is maintained, resulting in differentiation failure^17^. Cell proliferation using WST-8 was unchanged in T1KO mice; similarly, no difference was noted in the level of apoptosis. In addition, APCs, including dendritic cells and macrophages, were not increased in T1KO mice after allogenic BMT. Based on these results, we deduced that the mechanism underlying alleviating GVHD may be triggered by impaired differentiation of T lymphocytes into effector memory T cells. CD90.2 + cells were stimulated with PMA/ionomycin to examine the differentiation of CS and T lymphocytes. When cultured for 48 h, the differentiation failure became apparent, and effector memory cells for both CD4 and CD8 decreased in T1KO mice. In qPCR, the expression of IFN-γ and CCL2 decreased, and inflammatory signals decreased when SpMNCs were cultured with PMA/ionomycin.

Additionally, hepatic GVHD was significantly reduced in T1KO mice, and may be the cause of prolonged OS. In both T1KO and WT mice, the effecter memory T cells invaded the liver. PGs also interact with cell growth factors in the liver. T1KO mice deficient in EXT2, which can stop the glycan treatment of GAG, show a dominant increase in the amounts of CS and HS in the liver^18^. In addition, an increased abundance of CS and HS delayed the regenerative process from CCl_4_-induced hepatitis in EXT2^–/–^ mice^18^. These phenomena indicate that hepatic CS also demonstrates negative effects for reducing hepatic inflammation, and there is a possibility that hepatic CS reduction in T1KO may contribute significantly towards improved hepatic GVHD. Considering the effects of hematopoietic cell transplantation on hepatic GVHD, the homing ability of the hyaluronan receptor of donor CD44 lymphocytes was determined as CS competitively inhibits the binding of HA to CD44^9^. On the contrary, the production of HA in both Ito cells and fibroblasts increased in correlation with hepatic sclerosis^19^. Thus, binding effects between T cells and HA were examined, although our results showed no differences in T cells of T1KO and WT mice. However, T1KO T lymphocytes showed decreased production of anti-inflammatory cytokines and chemokines via qPCR analysis.

In conclusion, the reduction of CS expression in donor cells was shown to be associated with the alleviation of hepatic GVHD through the inhibition of T lymphocyte activation and differentiation, not by immune modulation of alloreaction. Hence, we postulate that CS may be an important therapeutic target of human GVHD, and warrants further investigations in the future.

## Methods

### Animals and ethics statement

T1KO mice, which were previously described, were maintained on the C57BL/6 background (H-2^b^)^12^. Balb/c (H-2^d^) mice were purchased from Japan SLC Inc. (Hamamatsu, Japan). All animal experiments in this study were performed with the approval of the Animal Ethics Committees of Niigata University (SA01134, SD01304) and performed in accordance with the relevant local and national guidelines and regulations. The study was also carried out in compliance with the ARRIVE guidelines.

### Transplantation of donor cells

BM-MNCs and SpMNCs were obtained as described in a previous study^20^. CD90.2^+^ cells were obtained from spleen cell preparations via positive selection using anti-CD90.2 microbeads (Miltenyi Biotec Inc. CA, USA). For transplantation, Balb/c mice (8–12 weeks old) were lethally irradiated (7 Gy) in a single fraction using an MBR-1605R (Hitachi power solutions, Ibaraki, Japan). BM-MNCs and SpMNCs were injected into the recipient mice through the tail vein at 6–8 h after irradiation. Murine GVHD scores were also measured as described in a previous study^21^.

### Flow cytometry

Flow cytometric analysis was performed on CytoFLEX (Beckman Coulter, CA, USA). To analyze liver infiltrating cells, hematopoietic cells were isolated by MACS Liver dissociation kit (Miltnyi Biotec Inc.) and Gentle MACS (Miltnyi Biotec Inc.). Antibodies were sourced from Biolegend: anti-CD3 phycoerythrin/Cyanine7 (PE/Cy7; 17A2), anti-CD4 phycoerythrin (PE; GK1.5), anti-B220 PE (RA3-6B2), anti-CD90.2 PE (30-H12), anti-CD11c PE/Cy7 (N418), anti-F4/80 fluorescein isothiocyanate (FITC; BM8), anti-NK1.1 Alexa Fluor 488 (PK136), anti-CD49b PE (DX5), anti-CD44 allophycocyanin (APC; IM7), anti-CD62L allophycocyanin/Cyanine7 (APC/Cy7 (MEL-14), anti-CD25 PE (PC61.5), anti-CD69 FITC (H1.2F3), anti-CD49d PE (MFR4.B), anti-CXCR3 APC (CXCR3-173), anti-PD-1 PE (29F.1A12), anti-KLRG-1 APC (2F1/KLRG1), anti-LFA-1 PE (H155-78), anti-LPAM-1 PE (DATK32), anti-H-2 Kb APC (AF6-88.5) and anti-CD8a FITC (53–6.7), anti-CD11b PE (M1/70) from BD Biosciences.

### Stimulation of T cells and apoptosis assay

CD90.2^+^ cells were enriched from spleen cell preparations via positive selection using anti-CD90.2 microbeads (Miltenyi Biotech). CD90.2^+^ cells or SpMNCs (2 × 10^5^) were cultured with both 2.5 ng/mL PMA (Sigma-Aldrich, MO, USA) and 0.5 μg/mL ionomycin (Sigma-Aldrich) for 24 or 48 h. For apoptosis assay, CD90.2^+^ cells were cultured for 48 h, and thereafter, cells were harvested by employing an Apoptosis detection Kit (Biolegend, San Diego, CA, USA) in accordance with the manufacturer’s instructions. Naïve/memory T cell ratio was also measured.

### HA binding analysis

Hyaluronan binding was measured using 1 mg/mL fluorescein-conjugated rooster comb hyaluronan, referred to here as FAHA-H1 (PG Research, Tokyo, Japan). FAHA-H1 and SpMNCs were reacted on ice for 1 h and analyzed with CytoFLEX.

### Cell proliferation assays

Cell viability was determined by WST-8 assay (Dojin Laboratory, Kumamoto, Japan) according to the manufacturer’s instructions. In brief, 2 × 10^5^ SpMNCs were cultured in a 96 well-plate for 48 h. To stimulate splenocyte proliferation, recombinant mouse IL-2 (mIL-2) (20 ng/mL; R&D Systems, Minneapolis, MN, USA) or anti-CD3/CD28 antibody-coated beads (Dynabeads mouse CD3/CD28 T-cell expander; Thermo Fisher Scientific, Hemel Hempstead, UK) or 2.5 ng/mL PMA and 0.5 μg/mL Ionomycin were added to the medium. The absorbance (450 nm) was measured 1 h after adding the cell-counting reagent, WST-8.

### Mixed lymphocyte reaction

A mixed lymphocyte reaction was performed using Balb/c (H-2^d^) as a stimulator and T1KO (H-2^b^) as a responder. Balb/c-SpMNCs were used for a stimulator, and these stimulator cells were irradiated with 30 Gy of gamma rays by a ^137^Cs irradiator using a PS-3000SB (PONY INDUSTRY Co., Ltd. Osaka, Japan). T1KO-CD90.2^+^ cells were enriched from spleen cell preparations via positive selection using anti-CD90.2 microbeads (Miltenyi Biotech) and used as a responder. The cells were cultured with 2 × 10^5^ stimulators and 1 × 10^6^ responders in medium with 10% FBS, and a cell proliferation assay was performed on day 7 using WST-8 assay (*Dojin* Laboratory, Kumamoto, *Japan*).

### RT-PCR analysis

Total RNA was extracted from SpMNCs using the RNeasy Mini kit (Qiagen, Hilden, Germany) according to the manufacturer's instructions. Gene mRNA levels were determined as described previously^22^. Interferon gamma (IFN-γ) (Mm01168134), C–C motif chemokine ligand 2 (CCL2) (Mm00441242), Tumor Necrosis Factor α (TNF-α) (Mm00443258), Interleukin (IL)-2 (Mm00434256), and GAPDH (Mm99999915) mRNA levels were determined via RT-qPCR using Taqman probes (Thermo Fisher SCIENTIFIC).

### Quantification of CS chains

GAGs from splenic cell preparations were prepared as described previously^23^ with slight modifications. Briefly, cells were homogenized in ice-cold acetone at least thrice and air-dried. The dried materials (acetone powders) were exhaustively digested with heat-activated actinase E [10% (w/w) of dried materials] in 0.1 M borate buffer, pH 8.0, containing 10 mM CaCl_2_, at 55 °C for 48 h. The digest was treated with 5% trichloroacetic acid, and the resultant acid-soluble fraction was adjusted to contain 80% ethanol. The resultant precipitate was dissolved in water and subjected to gel filtration on a PD-10 column (Cytiva, MA, USA) using water as an eluent. The flow-through fractions were collected, evaporated to dryness, and dissolved in water (crude GAG fractions).

An aliquot of the crude GAG sample was digested with a chondroitin sulfate-degrading enzyme, chondroitinase ABC (5 mIU, Seikagaku, Tokyo, Japan) in 50 mM Tris–HCl, and 60 mM sodium acetate, pH 8.0, at 37 °C for 2 h. The digests were derivatized with the fluorophore 2-aminobenzamide and were then analyzed by anion-exchange HPLC on a PA-G column (YMC, Kyoto, Japan) as described previously^24^. Identification and quantification of the resulting disaccharides were achieved by comparison with authentic unsaturated CS disaccharides (Seikagaku).

### Statistical analysis

Data were analyzed using unpaired Student *t* tests or variance (ANOVA) corrected for multiple testing. OS was analyzed via Mantel-Cox Log-rank test.* P*-values for specific comparisons were determined using GraphPad Prism (GraphPad Software, CA, USA). Further analysis is indicated in the figure legends. Each sample collection was performed at least twice.

### Supplementary Information

Supplementary Figures.

## Data Availability

The datasets generated during and/or analyzed during the current study are available from the corresponding author upon reasonable request.
