# Supplementary material for: Genetic manipulation resulting in decreased donor chondroitin sulfate synthesis mitigates hepatic GVHD via suppression of T cell activity
Source: Sci Rep. 2023 Aug 11;13:13098. doi: 10.1038/s41598-023-40367-3 (PMC10421903; doi:10.1038/s41598-023-40367-3)
Supplement: Supplementary file 1 — Supplementary Figures. [file 41598_2023_40367_MOESM1_ESM.docx]

**Genetic manipulation resulting in decreased donor chondroitin sulfate synthesis mitigates hepatic GVHD via suppression of T cell activity**

Suguru Tamura^1^, Hajime Ishiguro^1^, Tatsuya Suwabe^1^, Takayuki Katagiri^1^, Kaori Cho^1^, Kyoko Fuse^1^, Yasuhiko Shibasaki^1^, Tadahisa Mikami^2^, Takero Shindo^3^, Hiroshi Kitagawa^2^, Michihiro Igarashi^4^, Hirohito Sone^1^, Masayoshi Masuko^1^ and Takashi Ushiki^1,5,^*****

**Fig S1. Schematic representation of CSPGs and pathways of CS synthesis.**

CS is a type of GAG and a major component of ECM. The CS chain is bound to the serine residue of the core protein expressed on the surface of blood cells, including the lymphocyte surface, via a linker protein. Expression of CS GalNAc T1, the rate-limiting enzyme in CS synthesis, is knocked out in T1KO mice, resulting in decreased expression of CS in blood cells. Sulfate residues and sulfotransferases involved in the chain modifications are not included in this figure. This figure was created by modifying reference 4.

**Figure S2. Mixed lymphoid culture.**

Using Balb/c (H-2^d^) as stimulator and T1KO (H-2^b^) as responder, a mixed lymphocyte test was performed. SpMNCs were used, and stimulator cells were irradiated with 30 Gy of gamma rays. The cells were cultured with 8.0 × 10^5^ stimulators and 2.0 × 10^5^ responders in medium with 10% FBS, and a cell proliferation assay was performed on day 5 using WST-8. Stimulator: Balb/c SpMNCs; Responder: T1KO SpMNCs. n =3 in each group. This experiment was performed twice. Mean ± SD is shown with **p*<0.05 and ***p*<0.01 for comparison, one-way ANOVA with Tukey’s multiple comparisons test. CyA: cyclosporine 200 μg/mL.

**Figure S3. PD-1 expression in mice lacking T1.**

The SpMNCs were stimulated in culture in the presence of PMA/ionomycin. Expression of PD-1 before and after culture is shown. (A) Representative imaging of FACS analysis and (B) percentage of PD-1 expression in CD4 and CD8 T cells. n = 3 in each group. This experiment was performed thrice. Mean ± SD is shown with **p*<0.05, ***p*<0.01, ****p*<0.001, and *****p*<0.0001 for comparison, unpaired Student *t*-tests.
